# Beneficial Effects of *Lactobacillus plantarum* Strains on Non-Alcoholic Fatty Liver Disease in High Fat/High Fructose Diet-Fed Rats

**DOI:** 10.3390/nu12020542

**Published:** 2020-02-20

**Authors:** Eun-Jung Park, You-Suk Lee, Sung Min Kim, Gun-Seok Park, Yong Hyun Lee, Do Yeun Jeong, Jihee Kang, Hae-Jeung Lee

**Affiliations:** 1 Department of Food and Nutrition, Gachon University, Seongnam, Gyeonggi-do 13120, Korea; ejpark@gachon.ac.kr (E.-J.P.); ysleeyun@gachon.ac.kr (Y.-S.L.); kimsm127@gc.gachon.ac.kr (S.M.K.); 2 Institute for Aging and Clinical Nutrition Research, Gachon University, Seongnam, Gyeonggi-do 13120, Korea; 3 AtoGen Co., Ltd., Daejeon, 34015, Korea; gspark@atogen.co.kr (G.-S.P.); tppoc002@atogen.co.kr (Y.H.L.); jhkang@atogen.co.kr (J.K.)

**Keywords:** *Lactobacillus plantarum*, high-fat/high-fructose diet, non-alcoholic fatty liver disease

## Abstract

Emerging evidence suggests that probiotics are beneficial in non-alcoholic fatty liver disease (NAFLD). This study aimed to explore the effects of two *Lactobacillus plantarum* strains, ATG-K2 and ATG-K6 (isolated from Korean fermented cabbage), in a rat model of high fat/high fructose (HF/HF) diet-induced NAFLD. Rats with NAFLD were randomized into four groups (HF/HF diet control, (HC); HF/HF diet with silymarin, (PC); HF/HF diet with ATG-K2, (K2); and HF/HF diet with ATG-K6, (K6)) with healthy rats on a normal diet serving as the negative control. After treatment, histopathological and biochemical analyses of the blood and liver tissue were conducted. In addition, fecal microbiota was analyzed using the MiSeq platform. Compared with HC rats, K2 and K6 rats experienced significantly lower body weight gain, displayed decreased hepatic lipid accumulation, had lower serum levels of aspartate aminotransferase and alanine aminotransferase, and showed increased antioxidant enzyme activities. Moreover, de novo lipogenesis-related genes were downregulated following K2 and K6 administration. The fecal microbiota of K2 and K6 rats contained a higher proportion of Bacteriodetes and a lower proportion of Fimicutes than that of HC rats. Taken together, our results suggest that *L. plantarum* strains ATG-K2 and ATG-K6 are potential therapeutic agents for NAFLD.

## 1. Introduction

The increase in non-communicable metabolic diseases, such as chronic liver disease, type 2 diabetes, and heart disease, is linked to rising obesity rates [1]. Approximately 90% of patients with severe obesity are diagnosed with non-alcoholic fatty liver disease (NAFLD), 37% with non-alcoholic fatty hepatitis, and 10% with liver cirrhosis [2]. NAFLD, which is characterized by lipid accumulation in over 5% of hepatocytes in the absence of excessive alcohol consumption, is one of the most prevalent liver diseases [3,4]. The condition does not usually cause any symptoms in its early stage, but it can progress to serious liver diseases, including liver fibrosis, cirrhosis, and cancer [5].

Sugars, especially fructose, can activate hepatic lipogenesis programs that exacerbate NAFLD [6]. Fructose is processed almost exclusively by the liver, where it is mostly metabolized to triglycerides (TG) by de novo lipogenesis (DNL) [6,7]. Fructose-derived precursors act as nutritional regulators of the transcription factors, such as sterol regulatory element binding protein 1c (SREBP-1c) and CCAAT/enhancer-binding protein alpha (C/EBPα), which in turn regulate the expression of hepatic gluconeogenesis and DNL genes [6]. It follows that fructose intake increases liver gluconeogenesis and DNL and elevates blood glucose and TG levels in humans [8,9]

Another cause of NAFLD is oxidative stress. Fat deposition occurring in the liver results in lipid peroxidation, and it promotes a variety of responses such as inflammation and fibrosis. Thus, antioxidants (e.g., silymarin, vitamin E) are proposed as candidates for NAFLD treatment [10,11]; however, there are no standard pharmacological therapeutic agents for NAFLD yet. Recently, emerging evidence suggests that the gut-liver axis is strongly related to NAFLD. There are several reports published clinical trials applying probiotics on NAFLD patients [12]. *Lactobacillus bulgaricus* and *Streptococcus thermophiles* treatment improved liver aminotransferases levels in adult NAFLD patients [13]. In addition, probiotic capsules containing *Lactobacillus acidophilus*, *Bifidobacterium lactis*, *Bifidobacterium bifidum,* and *Lactobacillus rhamnosus*, as a supplement also decreased liver aminotransferases levels in obese children with NAFLD [14]. These results indicate that the intake of certain probiotics has a beneficial effect on NAFLD.

*Lactobacillus plantarum*, which plays a key role in the production of various fermented foods, is one of the most important species of lactic acid bacteria (LAB). *L. plantarum* is found in diverse environments, such as the soil and the human gut, and is considered a potential probiotic [15]. In addition, some strains of *L. plantarum* exert hepatoprotective effects by improving liver enzyme activity and reducing serum TG and total cholesterol (TC) levels [16,17]; however, the role of *L. plantarum* in NAFLD is still unclear.

Two novel *L. plantarum* strains, ATG-K2 (K2) and ATG-K6 (K6), have been recently isolated from fermented cabbage, a Korean traditional food [18]. K2 and K6 display bile salt hydrolase (BSH) activity, an ability to generate hydrogen peroxide (H_2_O_2_), immunomodulatory effects, and antimicrobial properties [18]. BSH activity is important for secondary bile salt metabolism, which may contribute to bile resistance of bacteria and benefit the host’s cholesterol metabolism by lowering bile salt concentration [19]. H_2_O_2_ produced by *L. plantarum* strains may help to kill opportunistic pathogens and have radical scavenging activity [20]. This indicates that K2 and K6 could be attractive biological agents for antimicrobial. Many supplements with antioxidant and anti-inflammatory properties are also effective in NAFLD. Thus, we hypothesized that K2 and K6 would have the potential to ameliorate NAFLD. In this study, we aimed to assess the effects of *L. plantarum* K2 and K6 on NAFLD using an in vivo rat model.

## 2. Materials and Methods

### 2.1. Preparation of L. plantarum Strains

The K2 and K6 *L. plantarum* strains were isolated from Korean fermented cabbage as part of a previous study [18] and deposited in Korean Collection for Type Cultures (KCTC; https://kctc.kribb.re.kr/En/Kctc.aspx). Based on average nucleotide identity analysis [21], K2 and K6 showed 96.6% and 99.16% of sequence identity with type strain ATCC 14917. Details of the general features of *L. plantarum* strains K2 and K6 are shown in Table S1. The strains were cultured in de Man–Rogosa–Sharpe (MRS) medium (Difco Laboratories, Detroit, MI, USA) at 37 °C for 16 h. Subsequently, K2 and K6 were collected by centrifugation at 8000 rpm for 15 min at 4 °C and washed three times with phosphate buffered saline (PBS). Finally, the cells were suspended in 2 volumes of 12.5% trehalose, 10% skim milk, and 0.125% carboxymethyl cellulose (w/v) solution and lyophilized using a FD8508 freeze-dryer (IlShinBioBase, Dongduchen, Korea). The colony-forming units (cfu) of dried K2 and K6 powder were quantified by count on MRS-agar plate. The freeze-dried powder was re-suspended in PBS before it was orally administered to rats.

### 2.2. Animals and Treatment

**Figure 1:**
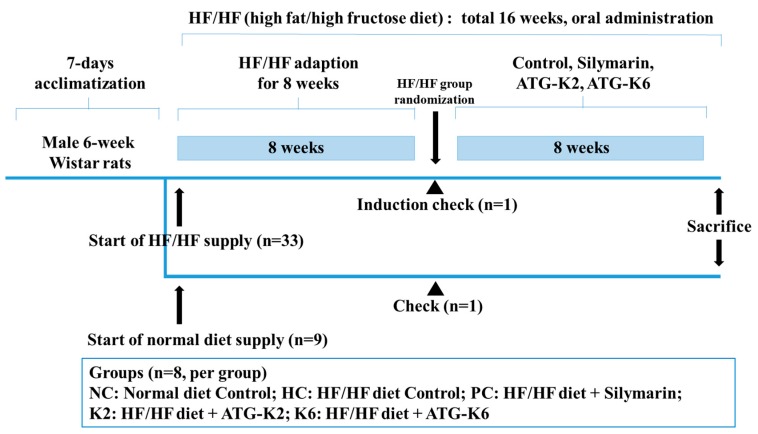
Schematic representation of the experimental schedule. HF/HF, high fat/high fructose.

A total of 42 male Wistar rats were purchased from Orient Bio Co. Ltd. (Seongnam, Korea) and housed under a controlled temperature (20–25 °C) and humidity (50–55%), with a 12 h/12 h light-dark cycle. Figure 1 shows schematic summary of the experimental schedule. The rats were allowed to acclimatize for seven days before they were randomly divided into two groups: healthy control group and NAFLD group. The rats were given free access to food and water, and were fed either a normal diet (D12450B; Research Diets, NJ, USA) or a high-fat (45%) diet (D12451, Research diet, NJ, USA) with 10% fructose in the drinking water (HF/HF). After eight weeks, one rat from each group was sacrificed to assess for the presence of fatty liver. Following NAFLD induction, the rats were randomized into five groups (*n* = 8 per group) according to their body weight: normal diet control (NC), HF/HF diet control (HC), HF/HF diet with 100 mg/kg silymarin (positive control, PC), HF/HF diet with K2 (5 × 10^8^ cfu, K2), and HF/HF diet with K6 (5 × 10^8^ cfu, K6). NC and HC rats were administered PBS, while PC, K2, and K6 rats were treated with the indicated materials using a sonde for an additional 8 weeks. Subsequently, all rats were fasted overnight and weighed before they were euthanized. Blood was collected by cardiac puncture. The blood was then centrifuged at 3000 rpm for 15 min at 4 °C (Combi-514R, Hanil Co. Ltd., Seoul, Korea) to extract the serum, which was stored at −80 °C until analysis. The rat livers were removed, rinsed with PBS, and stored immediately at −80 °C for future use. All animal procedures were approved by Gachon University for the care and use of the laboratory animals (ref. no. GIAUAC -R2019014).

### 2.3. Histological Analysis and Oil-Red O Staining

Samples of liver and epididymal tissue were fixed in 10% formalin (Sigma-Aldrich, St. Louis, MO, USA), embedded in paraffin wax, processed into 3–4 μm sections, and stained with hematoxylin and eosin (H&E). Gun-sucrose-fixed liver sections were stained with Oil-red O and observed under an Olympus Provis AX70 microscope (Olympus, Tokyo, Japan); images were captured with a Nikon DS-Ri2 camera (Nikon, Tokyo, Japan) using NIS-Elements BR 4.50.00 software (Tokyo, Japan).

### 2.4. Measurement of Serum Biochemistry Markers

Serum alanine aminotransferase (ALT), aspartate aminotransferase (AST), and alkaline phosphatase (ALP) levels were measured using ALT, AST, and ALP assay kits (Asanpharm, Hwaseong, Korea). Serum TG, TC, and high-density lipoprotein cholesterol (HDL-C) levels were quantified using TG, T-CHO, and HDL-CHO kits (Asanpharm, Hwaseong, Korea), respectively. Serum glucose levels were determined using a blood glucose meter kit (Handok, Seoul, Korea). Serum adiponectin and leptin levels were measured by an enzyme-linked immunosorbent assay (ELISA) using the appropriate kits (R&D system, Minneapolis, MN, USA). Colorimetric assay kits (BlueGene Biotech, Shanghai, China) were used to quantify serum levels of superoxide dismutase (SOD), glutathione peroxidase (GPx), and catalase (CAT). Data were calculated using the formulae described in each kit.

### 2.5. Measurement of Liver Biochemistry Markers

Hepatic samples were extracted according to the method of Folch, with some modifications. Liver tissue (0.1 g) was homogenized in a chloroform/methanol (2:1, v/v) solution, vortexed, and then centrifuged at 3000 rpm for 10 min at room temperature. After centrifugation, the bottom layer was carefully aspirated into a fresh test tube and evaporated to dryness under a stream of nitrogen. The dried lipid layer was weighed and re-dissolved in methanol prior to analysis. Hepatic TG and TC content was measured using the TG and T-CHO kits (Asanpharm, Hwaseong, Korea).

Malondialdehyde (MDA) levels were measured as previously described [22]. Briefly, MDA levels were quantified in the clear supernatant by measuring the absorbance at 532 nm, and expressed as nmol/g of liver tissue, with 1,1,3,3-tetraethoxypropane (Sigma-Aldrich, St. Louis, MO, USA) used as the external standard.

### 2.6. Quantitative Reverse Transcription PCR (qRT-PCR)

Total RNA was extracted from homogenized liver tissue using the easy-spin™ total RNA extraction kit (iNtRON Biotechnology, seongnam, Korea). RNA (50 ng) was converted to cDNA using the GoScript™ Reverse Transcriptase (Promega, Madison, WI, USA) and used as a template for real-time RT–PCR analysis with SYBR Green Master Mix (TaKaRa Bio, Otsu, Japan) using ABI QuantStudio 3 (Applied Biosystems, Foster City, CA, USA). Details of the primers used in the study are shown in Table 1. Target gene expression was normalized to that of Actb (β-actin).

### 2.7. Protein Extraction and Western Blotting

Proteins were extracted from the liver tissue samples using PRO-PREP™ Protein Extraction Solution (iNtRON Biotechnology, seongnam, Korea) containing the Halt™ phosphatase inhibitor cocktail (Thermo Scientific, Waltham, MA, USA). Following quantification with the Bicinchoninic acid assay (Thermo Scientific, Waltham, MA, USA), protein samples (30 μg) were separated by 6–10% sodium dodecyl sulfate polyacrylamide gel electrophoresis, then transferred onto polyvinylidene fluoride membranes. The membranes were blocked with 5% skim milk for 1 h at room temperature, and then washed and incubated with the indicated antibodies. Antibodies against SREBP-1c, fatty acid synthase (FAS), and CCAAT/enhancer-binding protein alpha (C/EBPα) were purchased from Santa Cruz Biotechnology (California, CA, USA). Antibodies against AMPK and phospho-AMPK (Thr172) were purchased from Cell Signaling Technology (Danvers, MA, USA). Antibodies against acetyl-CoA carboxylase (ACC), phospho-ACC (Ser79), carnitine palmitoyltransferase-1 (CPT1), and β-actin were obtained from Abcam (Cambridge, MA, USA). Horseradish peroxidase (HRP)-conjugated secondary antibodies were purchased from Promega. The signals were detected using the WEST-Queen™ RTS Western Blot Detection Kit (iNtRON Biotechnology, seongnam, Korea); the immunoreactive bands were visualized by chemiluminescence using the ImageQuant LAS 500 imager (GE Healthcare Life Sciences, Little Chalfont, UK).

### 2.8. Fecal Microbiota Profiling

Stool samples were collected from the rats at termination, using sterile tweezers. The metagenomic DNA was extracted using the QIAamp PowerFecal DNA Kit (QIAGEN, Germany). The quantity and quality of extracted DNA were measured using the Qubit 4 Fluorometer (Thermo Scientific, Waltham, MA, USA), NanoDrop One Microvolume UV-Vis Spectrophotometer (Thermo Scientific, Waltham, MA, USA), and agarose gel electrophoresis. The V3–V4 hypervariable regions of the bacterial 16S rRNA were amplified with unique 8 bp barcodes and sequenced on the Illumina MiSeq PE300 platform according to standard protocol [23]. The sequenced reads were analyzed using the Quantitative Insights into Microbial Ecology (QIIME) pipeline [24]. Briefly, sequences were filtered and clustered into operational taxonomic units (OTUs) at 97% sequence identity according to the SILVA 128 database [25] before weighted UniFrac distances matrices were calculated for principal coordination analysis (PCoA) [26].

### 2.9. Statistical Analysis

The data were expressed as mean ± standard deviation (*n* = 8 per group). One-way analysis of variance (ANOVA) with Duncan’s multiple range tests was used to perform multiple comparisons between the groups. All statistical analyses were carried out in SPSS version 25 (SPSS Inc., Chicago, IL, USA). *p*-values of < 0.05 were considered statistically significant.

## 3. Results

### 3.1. Effect of the L. plantarum K2 and K6 on Hepatic Steatosis and Body Weight in the Rat Model of HF/HF Diet-Induced NAFLD

**Figure 2:**
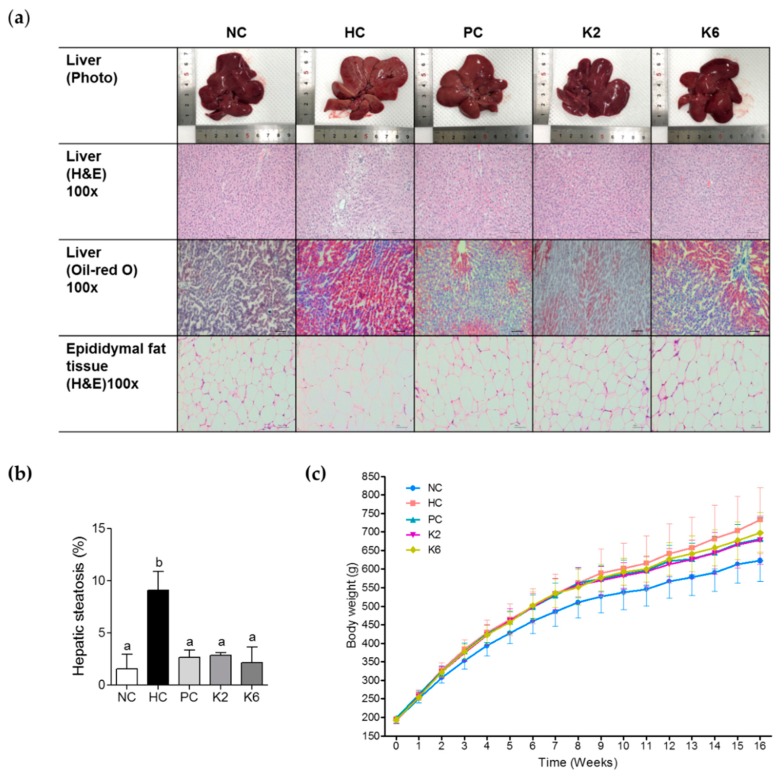
Effects of K2 and K6 on hepatic steatosis and body weight in rats with HF/HF-induced non-alcoholic fatty liver disease (NAFLD). (**a**) Histochemical and (**b**) morphometric analysis of liver samples and (**c**) body weight changes in rats on HF/HF diet with or without K2 and K6. NC, normal diet control; HC, HF/HF diet control; PC, HF/HF diet with silymarin; K2, HF/HF diet with ATG-K2; K6, HF/HF diet with ATG-K6. The data are presented as mean ± SD. Means with different letters (a, b) are significantly different, *p* < 0.05, whereas those with similar letters are not different. The groups were compared using one-way ANOVA followed by Duncan’s multiple range tests.

We first assessed hepatic steatosis in the rat model. Visual inspection of rat liver tissue following eight weeks of a HF/HF diet revealed altered color and lipid accumulation. To investigate the effect of *L. plantarum* K2 and K6 on hepatic fat accumulation, we assessed hepatic steatosis by visual examination, H&E staining, and Oil-red O staining after eight weeks of treatment (Figure 2a). H&E staining showed widespread lipid deposits in the HC group, with less deposits in the PC, K2, and K6 groups (Figure 2b). Oil Red O staining confirmed these findings. Moreover, H&E staining of epididymal fat tissue revealed that adipocyte size was smaller in the K2 and K6 groups than in the HC group.

As shown in Figure 2c and Table 2, final body weights of rats in the HC group were higher than those of rats in the NC group. In addition, higher weight gain was observed in HC rats than in NC rats. In contrast to HC feeding, K2 and K6 administration significantly suppressed body weight gain as well as PC groups.

### 3.2. The effects of L. plantarum K2 and K6 on Serum Biochemical Parameters

**Figure 3:**
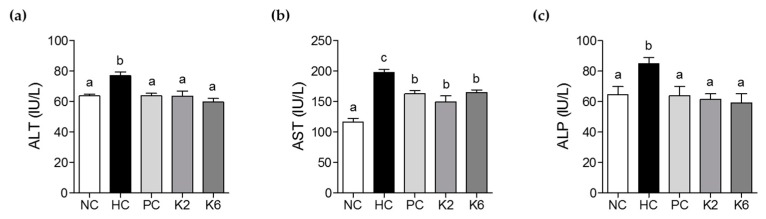
Effects of K2 and K6 on serum ALT, AST, and ALP levels in the rat model of HF/HF-induced NAFLD. (**a**) Serum ALT, (**b**) serum AST, and (**c**) serum ALP levels. NC, normal diet control; HC, HF/HF diet control; PC, HF/HF diet with silymarin; K2, HF/HF diet with ATG-K2; K6, HF/HF diet with ATG-K6; ALT, alanine aminotransferase; AST, aspartate aminotransferase; ALP, alkaline phosphatase. The data are presented as mean ± SD. Means with different letters (a, b, and c) are significantly different, *p* < 0.05, whereas those with similar letters are not different. The groups were compared using one-way ANOVA followed by Duncan’s multiple range tests.

**Figure 4:**
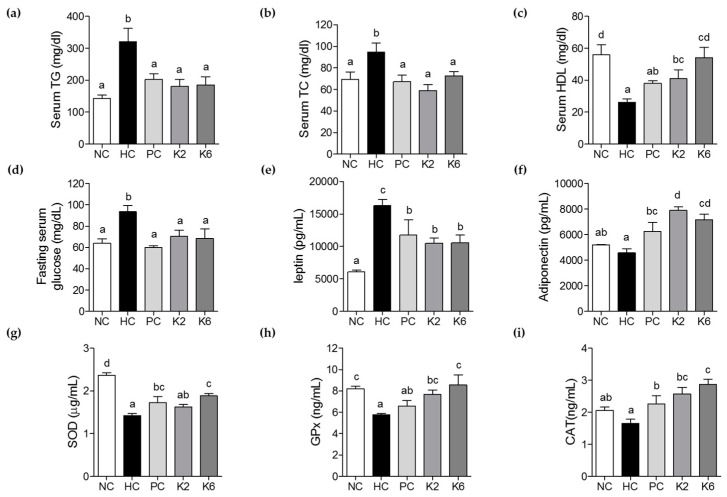
Effects of K2 and K6 on serum biochemical parameters in the rat model of HF/HF-induced NAFLD. (**a**) Serum TG, (**b**) serum TC, (**c**) serum HDL, (**d**) fasting serum glucose, (**e**) leptin, (**f**) adiponectin, (**g**) superoxide dismutase (SOD), (**h**) GPx, and (**i**) CAT levels. NC, normal diet control; HC, HF/HF diet control; PC, HF/HF diet with silymarin; K2, HF/HF diet with ATG-K2; K6, HF/HF diet with ATG-K6; TG, triglycerides; TC, total cholesterol; HDL, high-density lipoprotein; SOD, superoxide dismutase; GPx, glutathione peroxidase; CAT, catalase. The data are presented as mean ± SD. Means with different letters (a, b, c, and d) are significantly different, *p* < 0.05, whereas those with similar letters are not different. The groups were compared using one-way ANOVA followed by Duncan’s multiple range tests.

The activities of serum ALT, AST, and ALP were measured to evaluate the effect of K2 and K6 on liver function and damage. Serum ALT, AST, and ALP levels were significantly higher in the HC group than in the NC group (Figure 3a–c). Treatment with K2 and K6 significantly suppressed this effect, suggesting that both K2 and K6 have hepatoprotective properties. Levels of TG, TC, HDL-c, fasting serum glucose, adiponectin, and leptin were determined to assess the effects of K2 and K6 on serum lipid profile. As shown in Figure 4a–f, the HF/HF diet-induced increase in TG, TC, glucose, and leptin levels was significantly attenuated by treatment with K2 and K6. In addition, the decrease in HDL-c and adiponectin levels experienced by HC rats was prevented by administration of either strain. To assess the effect of K2 and K6 on antioxidant enzymes, we measured the activities of serum SOD, GPx, and CAT. The enzyme activities were markedly lower in the HC group than in the NC group; treatment with K2 and K6 suppressed this effect (Figure 4g–i).

### 3.3. The Effects of L. plantarum K2 and K6 on Hepatic Biochemical Parameters

**Figure 5:**
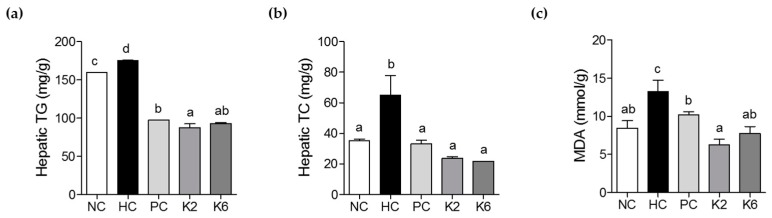
Effects of K2 and K6 on hepatic biochemical parameters in the rat model of HF/HF-induced NAFLD. (**a**) Hepatic TG, (**b**) TC, and (**c**) MDA levels. NC, normal diet control; HC, HF/HF diet control; PC, HF/HF diet with silymarin; K2, HF/HF diet with ATG-K2; K6, HF/HF diet with ATG-K6; TG, triglycerides; TC, total cholesterol; MDA, malondialdehyde. The data are presented as mean ± SD. Means with different letters (a, b, c, and d) are significantly different, *p* < 0.05, whereas those with similar letters are not different. The groups were compared using one-way ANOVA followed by Duncan’s multiple range tests.

TG and TC levels were measured in rat liver tissue to determine the effect of *L. plantarum* K2 and K6 on HF/HF diet-induced NAFLD. Hepatic TG levels were lower in K2- and K6-treated rats compared to untreated rats. There was a significant difference in hepatic TG levels between K2 and PC groups, but not between K2 and K6 groups (Figure 5a). Hepatic TC and MDA levels were significantly higher in HC rats than in NC rats. In contrast, they were reduced in K2- and K6-treated rats (Figure 5b). Similarly, MDA levels were higher in the HC group than in the NC group; this effect was also suppressed by treatment with K2 or K6 (Figure 5c).

### 3.4. The Effects of L. plantarum K2 and K6 on Lipogenesis-Related Gene Expression in the Liver

**Figure 6:**
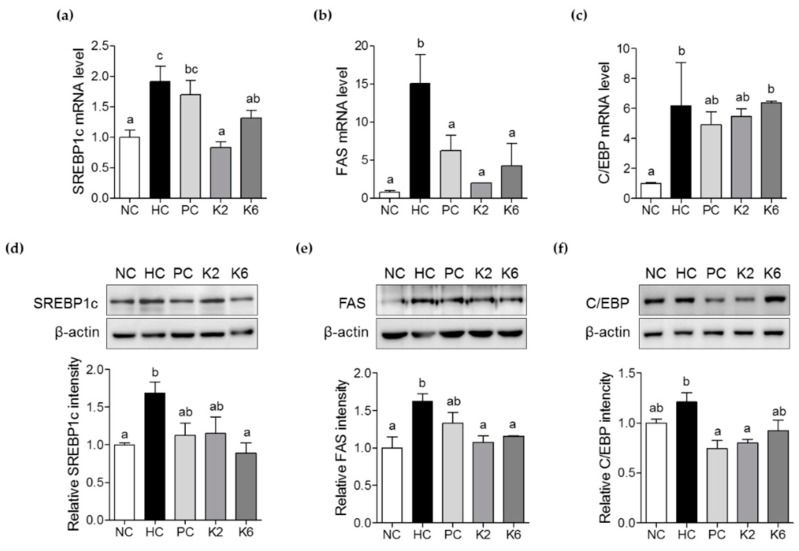
Effects of K2 and K6 on mRNA and protein expression of lipogenesis-related genes in the rat model of HF/HF-induced NAFLD. mRNA expression level of (**a**) SREBP-1c, (**b**) FAS, and (**c**) C/EBP in the liver. Western blot analysis of (**d**) SREBP-1c, (**e**) FAS, and (**f**) C/EBP expression in the liver. NC, normal diet control; HC, HF/HF diet control; PC, HF/HF diet with silymarin; K2, HF/HF diet with ATG-K2; K6, HF/HF diet with ATG-K6; SREBP-1c, sterol regulatory element binding protein 1c; FAS, fatty acid synthase; C/EBP, CCAAT/enhancer-binding protein. The data are presented as mean ± SD. Means with different letters (a, b) are significantly different, *p* < 0.05, whereas those with similar letters are not different. The groups were compared using one-way ANOVA followed by Duncan’s multiple range tests.

To investigate the molecular mechanisms underlying the effects of *L. plantarum* K2 and K6 on NAFLD, expression of lipogenesis-related genes was examined by RT-PCR and immunoblotting. SREBP-1c and FAS mRNA levels were significantly upregulated in the livers of HC rats compared to NC rats. mRNA expression of SREBP-1c and FAS was significantly lower in the K2 and K6 groups than in the HC group (Figure 6a,b). Similarly, mRNA levels of C/EBP were increased in the HC group, and tended to decrease in the PC and K2 groups. In line with this, immunoblot analysis showed that protein levels of SREBP-1c, FAS, and C/EBP were downregulated in K2 and K6 rats compared to HC rats (Figure 6d–f). These results indicate that the therapeutic effects of *L. plantarum* K2 and K6 on NAFLD are associated with downregulation of lipogenesis.

### 3.5. The Effects of L. plantarum K2 and K6 on Lipid Metabolism in the Liver

**Figure 7:**
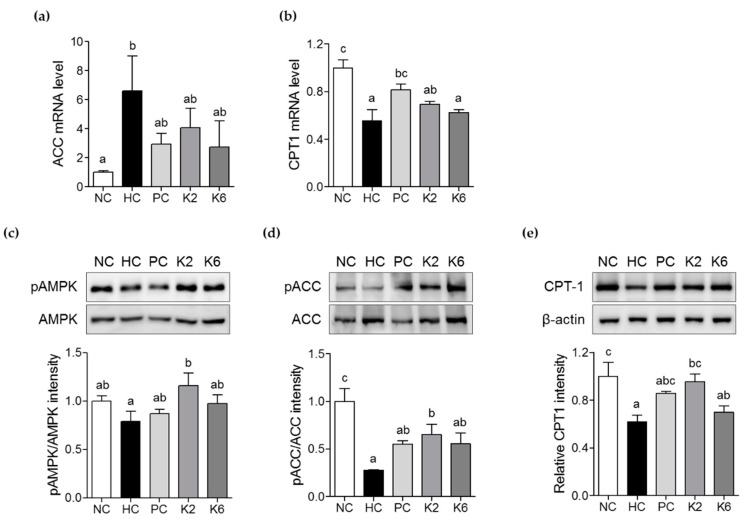
Effects of K2 and K6 on mRNA, protein, and phosphorylation levels of lipid metabolism-related markers in the rat model of HF/HF-induced NAFLD. mRNA expression levels of (**a**) ACC and (**b**) CPT-1 in the liver. Western blot analysis of (**c**) phosphorylated and total AMPK, (**d**) phosphorylated and total ACC, and (**e**) CPT-1 in the liver. Total AMPK and ACC were used as protein loading controls for phosphorylated AMPK and ACC, respectively. NC, normal diet control; HC, HF/HF diet control; PC, HF/HF diet with silymarin; K2, HF/HF diet with ATG-K2; K6, HF/HF diet with ATG-K6; AMPK, AMP-activated protein kinase; ACC, acetyl-CoA carboxylase; CPT-1, carnitine palmitoyltransferase-1. The data are presented as mean ± SD. Means with different letters (a, b, and c) are significantly different, *p* < 0.05, whereas those with similar letters are not different. The groups were compared using one-way ANOVA followed by Duncan’s multiple range tests.

To evaluate the effects of *L. plantarum* K2 and K6 on hepatic lipid metabolism, expression of genes involved in fatty acid oxidation, such as ACC and CPT-1, was measured by qRT-PCR. Expression of ACC mRNA was lower in K2- and K6- treated rats than in HC rats. There were no significant differences in ACC expression between the PC, K2, and K6 groups (Figure 7a). CPT-1 mRNA levels were also higher in the K2 and K6 groups than in the HC group (Figure 7b). To investigate whether *L. plantarum* K2 and K6 activates AMPK signaling, we performed an immunoblot analysis. As shown in Figure 7d–e, phosphorylation levels of both markers were enhanced following treatment with K2 or K6. CPT-1 protein expression was also higher in K2- and K6-treated than in in the HC group (Figure 7f).

### 3.6. The Effects of L. plantarum Administration on Fecal Microbiota Composition

**Figure 8:**
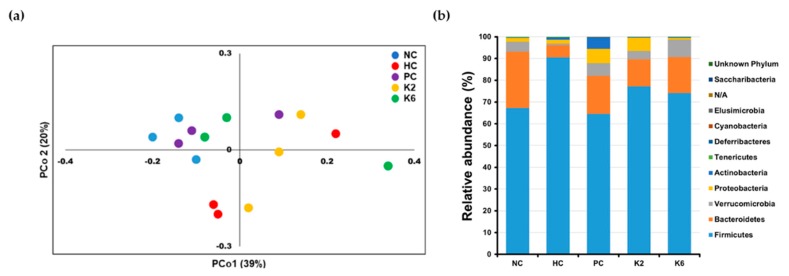
Fecal microbial diversity in each group. (**a**) Principal coordinates analysis (PCoA) plot using weighted UniFrac distance matrix; (**b**) Bar plot of relative bacterial phylum abundance. Each bar represents results of three pooled fecal samples. NC, normal diet control; HC, HF/HF diet control; PC, HF/HF diet with silymarin; K2, HF/HF diet with ATG-K2; K6, HF/HF diet with ATG-K6.

**Figure 9:**
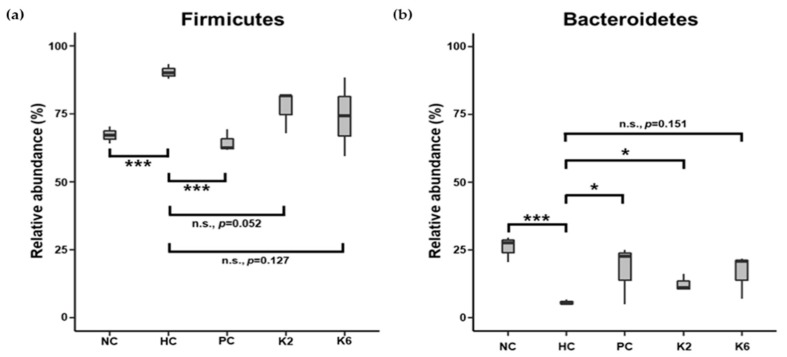
Relative abundance of phyla (**a**) Firmicutes and (**b**) Bacteroidetes in each group. Each box plot represents results of three pooled fecal samples. NC, normal diet control; HC, HF/HF diet control; PC, HF/HF diet with silymarin; K2, HF/HF diet with ATG-K2; K6, HF/HF diet with ATG-K6. * *p* < 0.05, *** *p* < 0.005, n.s., not significant versus the HC group. Student’s t-test was used to compare the group means.

**Figure 10:**
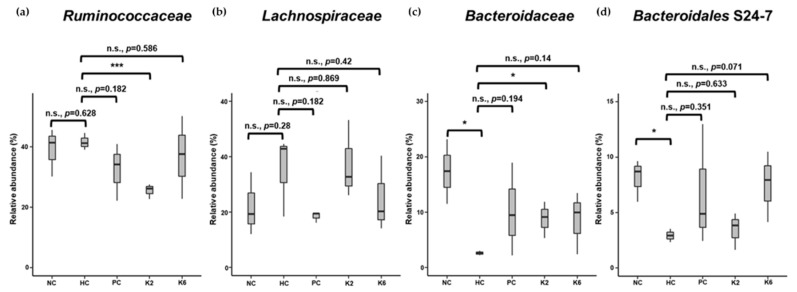
Relative abundance of family (**a**) *Ruminococcaceae*, (**b**) *Lachnospiraceae*, (**c**) *Bacteroidaceae*, and (**d**) *Bacteroidales* S24-7 in each group. Each box plot represents results of three pooled fecal samples. NC, normal diet control; HC, HF/HF diet control; PC, HF/HF diet with silymarin; K2, HF/HF diet with ATG-K2; K6, HF/HF diet with ATG-K6. * *p* < 0.05, *** *p* < 0.005, n.s., not significant versus the HC group. Student’s t-test was used to compare the group means.

To examine the effects of *L. plantarum* species on the gut microbial community, the bacterial composition of three pooled fecal samples from each experimental group was analyzed. The beta-diversity was examined through PCoA using the weighted UniFrac distance matrix. The microbiota of the HC group was clearly separated from those of other groups (Figure 8a). Mirroring the PCoA result, the microbiota of the PC, K2, and K6 groups had a relatively similar composition to that of the NC group, while the HC group showed a different bacterial community structure (Figure 8b and Table S1) with a higher abundance of Firmicutes (90.5%) and lower abundance of Bacteroidetes (5.6%) than in the other groups. NC, PC, K2, and K6 rats displayed 67.2%–77.1% abundance of Firmicutes and 12.4%–25.8% abundance of Bacteroidetes. Significant differences in bacterial populations were confirmed using student’s *t*-test (Figure 9). K2- and K6-treated rats showed similar changes in bacterial community structure as NC and PC rats. At the family level, *Ruminococcaceae* (phylum Firmicutes) and *Lachnospiraceae* (phylum Firmicutes) were more abundant, while *Bacteroidaceae* (phylum Bacteroidetes) and *Bacteroidales* S24-7 (phylum Bacteroidetes) were less abundant in the HC group than in the other groups (Figure 10).

## 4. Discussion

Probiotics have been used traditionally for gastrointestinal health in the form of yogurt and others [27]. Most of the immune cells against disease are produced in the intestine, therefore there is a growing interest in intestinal health due to probiotic intake. This interest extends to the improvement of various inflammatory diseases. Particularly, probiotic treatment is an effective and safe approach to reversing metabolic abnormalities observed not only in obesity [28,29], but also in NAFLD [30]. In this study, treatment with two *L. plantarum* strains, K2 and K6, improved HF/HF diet-induced NAFLD in rats by regulating liver function, serum lipid levels, and lipogenesis via the AMPK pathway. Our literature investigation confirmed that several probiotics administered alone or in combination, have improvement effects on liver function in vivo [17,31]. *L. plantarum* NCU116, isolated from pickled vegetables, has indicated the effect amelioration of NAFLD by lowering the cholesterol property [17]. Another study also showed that cocktail of probiotics (*L. acidophilus*, *L. plantarum*, *Bifidobacterium*, and *Bacillus subtilis* strains) affected host adipose tissue hormone levels such as leptin, resistin, and hepatic function markers [31]. These results provide the validity of our results, suggesting that our results will contribute to discovering ameliorative action mechanisms of probiotics against NAFLD. 

Measurement of liver function-related enzymes, such as ALT, AST, and ALP, can detect damage to the hepatocellular and biliary tracts [32]. Increased activity of ALT and AST can indicate NAFLD [33]. In particular, ALT is a hepatocyte-specific enzyme whose elevated serum levels directly reflect liver damage [32,34]. Our results showed that serum ALT levels were high in rats in the HC group, and low in rats in the K2- and K6-treated groups. In a previous study, *L. paracasei* Jlus66 isolated from natural fermented milk attenuated serum ALT activity in a rat model of high fat diet-induced NAFLD [30]. Taken together, these results suggest that probiotics isolated from fermented foods could exert protective effects on NAFLD by reducing ALT activity.

Increased free radical load in NAFLD can affect β-oxidation to enhance the production of reactive oxygen species (ROS) [35]. Accumulation of ROS causes oxidative stress, resulting in protein carbonylation and DNA damage [36]. MDA is produced by lipid peroxidation and considered a marker oxidative stress. In addition, SOD acts as a defender of oxidative stress that changes oxygen radicals to hydrogen peroxide and dioxygen [37]. In this study, the enzyme activity of SOD, GPx, and CAT was measured in the serum to assess the antioxidant effects of K2 and K6. Both K2- and K6-treated rats displayed higher antioxidant activities than HC rats. MDA levels were decreased in the K2 and K6 group, these results were similar to other study using *L. plantarum* NA 136 [16]. Our results demonstrate that K2 and K6 have the potential to be developed as hepatoprotective agents as indicated by higher antioxidant activities on the NAFLD rat model.

Nrf2 plays a crucial role in cellular defenses against oxidative stress by regulating expression of antioxidant enzymes [38]. Normally, Keap1 binds Nrf2 to sequester it in the cytoplasm. Once cells are exposed to the oxidative stress, Nrf2 is released from Keap1 and translocates into the nucleus. Once in the nucleus, Nrf2 dimerizes with Maf and stimulates the expression of antioxidant stress genes [39]. A previous study showed that CAT promotes Nrf2 translocation and targets its downstream genes, such as HO-1 gene, to trigger the Nrf2 defense system [40]. Reportedly, treatment with *L. plantarum* NA136 increased CAT expression, which was attenuated in the HFD/F group [16]. This in turn triggers Nrf2 translocation to regulate expression of cellular antioxidant enzymes, including SOD and HO-1. *Bacillus amyloliquefaciens* SC06 has also been reported to increase the level of CAT and activate the Nrf2/Keap1 signaling pathway to boost cellular antioxidant status [41]. Further studies are required to confirm the involvement of Nrf2/Keap1 signaling in *L. plantarum* K2- and K6-induced effects. 

NAFLD is closely related to insulin resistance in the liver [42]. Excessive glucose levels and insulin release in HF/HF diet can promote DNL via hepatic transcription factors, such as SREBP-1. Activated by downstream signaling, SREBP-1 increases FAS expression [43], which is involved in fatty acid synthesis. In NAFLD, FAS stimulates fatty acid accumulation in hepatocytes. In this study, lipid metabolism-associated pathways, such as leptin, adiponectin, SREBP-1, and ACC signaling, were restored by *L. plantarum* K2 and K6, which in turn decreased accumulation of TG and TC in the serum, resulting in weight loss. Adipose tissue can release free fatty acids to the liver, where the levels of leptin, adiponectin are altered, decreasing cholesterol levels in the blood and dampening anti-inflammatory processes mediated by cytokines such as TNF-α and IL-6 [44]. The results of the present study, and of our previous investigation of anti-inflammatory effects of *L. plantarum* K2 and K6 [18] suggest that the therapeutic effects of probiotics are mediated by regulation of insulin-, inflammation-, and lipogenesis-associated pathways. Moreover, these changes in serum lipid biomarkers by K2 and K6 are thought to also be effective in reducing the fat accumulation, similar to the function of probiotics such as *L. gasseri* BNR17 [45].

Oral administration of *L. plantarum* strains may alter intestinal and fecal microbiota [46]. Treatment with *L. plantarum* K2 or K6 induced similar changes to the composition of fecal microbiome. Although not all results reached statistical significance, administration of either K2 or K6 induced a shift in the microbial community that resulted in an increased abundance of Bacteroidetes, (including family *Bacteroidaceae* and *Bacteroidales* S24-7) and decreased abundance of Firmicutes, with respect to the untreated rats. Further studies are required to identify the active substances of K2 and K6 strains, modulation of Bacteroidetes population through oral administration of *Lactobacillus,* links to progression of fatty liver and NAFLD [47,48]. Interestingly, fecal microbiota of the silymarin-treated rats (positive control) also showed an increased proportion of Bacteroidetes and decreased proportion of Firmicutes. High relative abundance of Bacteroidetes in the gut microbiota correlates with liver health [49]; thus, *L. plantarum* K2 and K6 are potential probiotics owing to their ability to boost Bacteroidetes levels.

## 5. Conclusions

In the present study, *L. plantarum* strains K2 and K6 ameliorated HF/HF diet-induced NAFLD by regulating liver function and expression of antioxidant enzymes and lipogenesis-related genes. In addition, both strains were able to improve the composition of fecal microbiota. Our results provide a basis for the development of novel therapeutic strategies for NAFLD.

## Figures and Tables

**Table 1:** Primer sequences used for qRT-PCR.

Gene	Forward (5′-3′)	Reverse (5′-3′)
SREBP-1c	CCC TGC GAA GTG CTC ACA A	GCG TTT CTA CCA CTT CAG GTT TCA
FAS	GCT GCT ACA AAC AGG ACC ATC AC	TCT TGC TGG CCT CCA CTG AC
C/EBPα	GCC AAG AAG TCG GTG GAT AA	CCT TGA CCA AGG AGC TCT CA
ACC	CAA TCC TCG GCA CAT GGA GA	GCT CAG CCA AGC GGA TGT AGA
CPT-1	CCA TCT CTT CTG CCT CTA TGT	GTC AGG GTT TTT CTC AAA GTC
β-actin	GAT TAC TGC CCT GGC TCC TA	TCA TCG TAC TCC TGC TTG CT

SREBP-1c, Sterol regulatory element binding protein 1c; FAS, Fatty acid synthase; C/EBPα, CCAAT/enhancer-binding protein alpha; ACC, Acetyl-CoA carboxylase; CPT-1, Carnitine palmitoyltransferase-1.

**Table 2:** Body weight of rats in each groups (g).

Groups	Initial Body Weight	Body Weight after Randomization (A)	Terminal Body Weight (B)	Body Weight Gain (B-A)
NC	194.0 ± 8.4	485.5 ± 38.9 ^a^	622.8 ± 55.6 ^a^	137.2 ± 30.8 ^a^
HC	194.0 ± 7.6	522.4 ± 29.5 ^b^	733.3 ± 87.2 ^b^	210.9 ± 63.1 ^b^
PC		522.4 ± 28.6 ^b^	680.4 ± 57.5 ^ab^	158.0 ± 35.9 ^a^
K2		521.9 ± 31.1 ^b^	677.9 ± 64.8 ^ab^	156.0 ± 37.7 ^a^
K6		523.6 ± 27.1 ^b^	697.1 ± 55.6 ^b^	173.5 ± 30.9 ^a^

NC, Normal diet control; HC, high fat/high fructose (HF/HF) diet control; PC, HF/HF diet with silymarin; K2, HF/HF diet with ATG-K2; K6, HF/HF diet with ATG-K6. The data are presented as mean ± SD. Means with different letters (a, b) are significantly different, *p* < 0.05, whereas those with similar letters are not different. The groups were compared using one-way ANOVA followed by Duncan’s multiple range tests.

## Supplementary Materials

The following are available online at https://www.mdpi.com/2072-6643/12/2/542/s1, Table S1. ANI values for *Lactobacillus plantarum* strains. Tables S2–S4. Fecal Microbial Community Structure results.
